# Epidemiological and Clinical Characteristics of Pediatric Acute Drug Intoxications: A Retrospective Analysis

**DOI:** 10.3390/children12010044

**Published:** 2024-12-30

**Authors:** Cristina Elena Singer, Renata-Maria Văruț, Maria Singer, Simona Cosoveanu, Jaqueline Abdul Razzak, Madalina Elena Popescu, Simina Gaman, Ileana Octavia Petrescu, Cristina Popescu

**Affiliations:** 1 Department of Mother and Baby, University of Medicine and Pharmacy of Craiova, 200349 Craiova, Romania; cristina.singer@umfcv.ro (C.E.S.); simona.cosoveanu@umfcv.ro (S.C.); jaquelineabdulrazzak90@gmail.com (J.A.R.); aleca.madalina95@gmail.com (M.E.P.); ileana.petrescu@umfcv.ro (I.O.P.); 2 Research Methodology Department, Faculty of Pharmacy, University of Medicine and Pharmacy of Craiova, 200349 Craiova, Romania; 3 Dermatology Department, Central Military Hospital, 010825 Bucharest, Romania; maria.singer1897@gmail.com; 4 Department I, Faculty of Dental Medicine, University of Medicine and Pharmacy of Craiova, 200349 Craiova, Romania; simina.gaman@umfcv.ro; 5 County Hospital Craiova, Department of Anatomy, University of Medicine and Pharmacy, Discipline of Anatomy, 200349 Craiova, Romania; cristina.popescu@umfcv.ro

**Keywords:** pediatric acute drug intoxications, poisoning severity score, accidental and intentional ingestions, epidemiology of intoxications, public health strategies

## Abstract

Background/Objectives: Acute drug intoxications (ADIs) are a significant concern in pediatric healthcare, contributing to both accidental and intentional morbidity. This study aimed to analyze the demographic, clinical, and therapeutic characteristics of pediatric ADI cases to identify trends and inform preventive strategies. Methods: This retrospective study included 120 cases of pediatric ADI admitted to the Second Pediatric Clinic of Craiova County Emergency Clinical Hospital in 2022 and 2023. The inclusion criteria encompassed children aged 0–17 years with confirmed pharmaceutical intoxications. Cases involving mixed poisonings or non-pharmaceutical substances were excluded. Clinical severity was classified using the Poisoning Severity Score (PSS). Data on demographics, substances involved, clinical presentations, interventions, and outcomes were analyzed. Results: The majority of cases occurred in females (73.3%) and urban residents (77.5%). Accidental intoxications were prevalent in children aged 1–5 years (45%), while intentional ingestions were common in adolescents (47.5%). The most frequently implicated substances included antibiotics (46.7%), benzodiazepines (20.8%), and acetaminophen (15.8%). Severity was classified as mild (44.2%), moderate (26.6%), or severe (29.2%), while treatment primarily included supportive care, intravenous fluids (62.5%), and antidotes (35.8%). Severe cases required respiratory support in 29.2% of the instances. Hospitalization duration significantly decreased from 2022 (3.8 ± 1.9 days) to 2023 (2.3 ± 0.9 days) (*p* < 0.05), and no fatalities were recorded. Conclusions: Pediatric ADIs predominantly involve accidental ingestions in young children and intentional overdoses in adolescents. Targeted public health strategies, such as parental education, adolescent mental health support, and improved rural healthcare access, are essential to reduce incidence and severity. These findings underscore the need for focused prevention and optimized clinical management.

## 1. Introduction

Acute drug intoxication (ADI) in children is a significant global health concern, representing a leading cause of pediatric emergencies and hospital admissions. The World Health Organization defines acute intoxication as “a transient condition following the administration of alcohol or other psychoactive substances, resulting in disturbances in the level of consciousness, cognition, perception, affect, or behavior, or other psychophysiological functions and responses” [1]. In pediatric populations, ADI often results from accidental ingestions, given children’s natural curiosity and tendency to explore their environment orally. However, in adolescents, intentional ingestions, frequently associated with self-harm or suicide attempts, are more prevalent [2]. The global burden of ADI is further reflected in its incidence and severity. A study conducted across several regions reported that ADI accounted for approximately 10% of all pediatric emergency room visits, with higher prevalence rates in urban settings compared to rural areas [3]. Furthermore, accidental ingestions in children aged 1–5 years represent 60–80% of all ADI cases in this age group, while adolescents aged 10–17 years exhibit intentional ingestion rates of nearly 40%, often linked to psychiatric conditions such as depression and anxiety [4,5]. The spectrum of substances involved in pediatric intoxications is broad, encompassing pharmaceuticals, household products, and environmental toxins. Pharmaceuticals, particularly analgesics, sedatives, and antidepressants, are frequently implicated. For example, benzodiazepines, acetaminophen, and antibiotics have been identified as the leading causes of ADI in multiple studies [6,7]. Acetaminophen intoxication poses a significant challenge due to its potential to cause acute liver failure, a condition known as hepatotoxicity. Hepatotoxicity refers to liver damage resulting from exposure to toxic substances, which can lead to life-threatening complications if not promptly treated. Among these, acetaminophen intoxication poses a significant challenge due to its potential to cause acute liver failure, accounting for 10–20% of pediatric liver transplant cases globally [8]. In contrast, benzodiazepines are often associated with central nervous system depression, highlighting the need for timely diagnosis and intervention. The clinical manifestations of ADI are diverse, ranging from mild symptoms such as nausea and dizziness to severe outcomes such as seizures, respiratory depression, and coma. The severity is influenced by factors such as the type and quantity of the ingested substance, the child’s age and weight, and the timeliness of medical intervention [9]. While mortality rates for pediatric ADI remain low (<2%), morbidity associated with delayed treatment or severe intoxications can be substantial, including long-term neurological sequelae in severe cases [10]. Despite advances in medical care, ADI remains a persistent challenge, particularly in low- and middle-income countries, where access to healthcare and public health education is limited. Preventive measures, including stricter regulations on drug storage and packaging, public education campaigns, and awareness initiatives targeting caregivers and adolescents, are critical for reducing the incidence of pediatric poisonings. Recent studies also emphasize the role of community-based poison prevention programs and telemedicine platforms in providing rapid consultation and reducing adverse outcomes [11,12,13,14]. Our study addresses key gaps in pediatric ADI research by focusing on underexplored areas, such as the role of antibiotics in intoxications, stratification of cases by clinical severity, and the influence of socio-demographic factors like urban versus rural residency. Unlike previous studies, which often analyze narrow time frames or specific drug categories, our research provides a multi-year, comprehensive analysis, highlighting trends, disparities, and actionable insights for prevention and management. The increasing prevalence of ADIs among pediatric populations underscores the complex interplay between environmental, socioeconomic, and behavioral factors. Urbanization, with its concomitant increase in access to pharmaceuticals, coupled with inadequate medication storage practices, significantly elevates the risk of accidental intoxications in younger children. In contrast, adolescents face a different set of challenges, including psychosocial stressors, peer influence, and access to potentially harmful substances, which often result in intentional poisonings. Cultural and familial dynamics also play a critical role, as studies suggest that single-parent households or families with a history of mental health disorders may exhibit higher rates of pediatric poisonings. Moreover, seasonal variations, with peaks during school holidays, highlight the importance of consistent supervision and preventive measures during these vulnerable periods. By examining these multifaceted determinants, this study aims to contribute to the understanding of ADI patterns and inform evidence-based interventions tailored to specific risk groups.

## 2. Materials and Methods

This retrospective study analyzed 120 pediatric cases of ADI admitted to the Second Pediatric Clinic of Craiova County Emergency Clinical Hospital in 2022 and 2023. Data were collected from patient medical records, including demographics (age, gender, and urban/rural origin), substances involved, clinical manifestations, treatment modalities, and outcomes. The inclusion criteria were patients aged 0–17 years with a confirmed diagnosis of ADI, while cases with incomplete data or mixed poisoning involving non-pharmaceutical substances were excluded. The severity of intoxication was classified as mild, moderate, or severe based on clinical presentations, laboratory findings, and the need for specific interventions. This classification follows the Poisoning Severity Score (PSS), a standardized tool in toxicology that categorizes poisoning cases based on symptoms and their impact on vital functions, aiding in assessing prognosis and guiding treatment. The classification aligned with the Poisoning Severity Score (PSS), a validated tool in toxicological research, which allows for grading based on clinical outcomes:

- Mild: minimal symptoms requiring only observation or supportive care.
- Moderate: symptoms requiring medical intervention such as antidote administration or monitoring in a general ward.
- Severe: life-threatening symptoms necessitating intensive care, such as respiratory or cardiac support.

Statistical analyses were performed using SPSS version 26.0. Descriptive statistics summarized the demographic and clinical data, while inferential statistics, including chi-square tests for categorical variables and t-tests for continuous variables, were used to assess differences between the years. A *p*-value of <0.05 was considered statistically significant. Ethical approval for this study was obtained from the hospital’s ethics committee, and patient anonymity was ensured throughout the data collection and analysis.

## 3. Results

The gender distribution analysis indicated a significant predominance of female patients, who accounted for 73.3% of the cases across the two study years, yielding a female-to-male ratio of 2.75. Regarding age distribution, the frequency of ADI was lowest among infants aged 0–1 year (2.5%) and children aged 6–9 years (5%). In contrast, the highest frequencies were observed in the 1–5-year age group (45%) and the 10–17-year age group (47.5%). Notably, in 2022, accidental intoxications were most prevalent in the 1–5-year age group (53.2%), whereas in 2023, voluntary poisonings predominated in adolescents aged 10–17 years, with a frequency of 60.97%. These findings underscore the age-specific patterns of ADI, with younger children primarily affected by accidental exposure and adolescents by intentional ingestion (Table 1).

The distribution of cases by age showed statistically significant differences between years for the 1–5 age group (*p* = 0.014) and adolescents aged 10–17 years (*p* = 0.038), with a notable increase in intentional ingestions among adolescents in 2023. Gender and environment distributions, however, did not differ significantly between years (*p* > 0.05). No statistically significant differences were found between 2022 and 2023 in gender distribution (*p* = 0.532) or living environment (urban vs. rural; *p* = 1.000). These findings indicate consistent demographic patterns across both years. Furthermore, the seasonal trends in voluntary adolescent ingestions were consistent across the two years (*p* = 0.999), with no significant variation in the distribution of cases across spring, summer, autumn, and winter. These findings indicate that voluntary adolescent ingestions are not strongly influenced by geographical or seasonal factors, emphasizing the need to address the psychosocial drivers that may underlie these patterns.

The analysis of the distribution of cases by origin demonstrates that the majority of intoxicated children (77.5%) were from urban areas. This finding is particularly notable given that the majority of pediatric admissions to the clinic during the study period were from rural regions. This contrast highlights a significant discrepancy between the prevalence of ADI in urban versus rural populations, suggesting that urban residency may be a key risk factor, potentially linked to increased access to pharmaceuticals and reduced supervision (Table 2). The majority of cases in both years were from urban regions, with females significantly outnumbering males (*p* < 0.05, odds ratio 2.75). No significant year-to-year variations in urban/rural distributions were noted.

The majority of patients were from low-income families (45.8%), followed by those from middle-income (42.5%) and high-income households (11.7%). Among the families, most parents were both employed (71/120, 59.2%), while a smaller proportion had only one parent employed (27.5%) or both parents unemployed (13.3%). The education level of parents revealed a near-equal distribution between primary (40.8%) and secondary education (39.2%), with a smaller proportion of families where parents had tertiary education (20%). Household size varied, with most children living in families with 1–2 siblings (50.8%), while fewer had no siblings (9.2%) or 3 or more siblings (40%). Larger families may face additional caregiving challenges, potentially increasing the risk of accidental exposure. Medication storage practices showed that 55.8 households stored medications in secured locations, whereas 44.2 households stored medications in unsecured locations. This highlights a critical risk factor for accidental drug intoxications. A minority of families (16.7%) reported a history of chronic illness, while the majority (83.3%) did not have such a history (Table 3).

The statistical analysis revealed no significant differences between the years 2022 and 2023 for socioeconomic status, parental employment status, parental education level, number of siblings in the household, medication accessibility, or history of chronic illness in the family. Specifically, the distribution of socioeconomic status remained consistent across the two years, with the proportions of low-income, middle-income, and high-income households showing no statistically significant variation (*p* = 0.893). Similarly, parental employment patterns did not differ significantly, as the percentages of families where both parents were employed, only one parent was employed, or both parents were unemployed were stable over the study period (*p* = 0.934).

Parental education levels also exhibited no significant year-to-year changes, with a near-equal distribution of primary, secondary, and tertiary education levels observed across the two cohorts (*p* = 0.993). The number of siblings in the household, categorized as no siblings, one to two siblings, and three or more siblings, demonstrated no significant variations between the two years (*p* = 0.771). Furthermore, the accessibility of medications within households, whether stored in secured or unsecured locations, remained consistent, showing no statistical differences between 2022 and 2023 (*p* = 0.879). Lastly, the prevalence of a positive family history of chronic illness compared to no history of chronic illness did not vary significantly across the two study years (*p* = 0.731).

**Figure 1:**
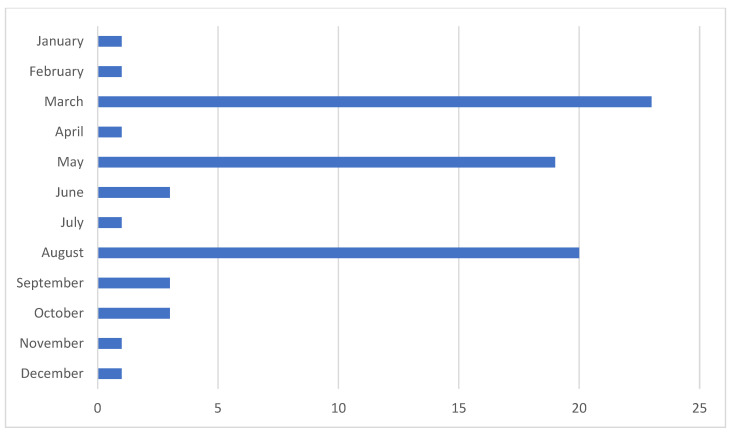
ADI frequency by month in 2022.

The distribution of ADI cases by calendar month in 2022 revealed notable variations in incidence. Higher rates were observed in March (23 out of 79 cases), August (20 out of 79 cases), and May (19 out of 79 cases), indicating peak months of ADI occurrence. Conversely, lower incidences were recorded in June, September, and October, with only 3 cases reported in each of these months. These findings highlight potential seasonal trends in ADI, which may be influenced by factors such as changes in routine, school holidays, and levels of supervision. Figure 1 provides a visual representation of these monthly variations.

**Figure 2:**
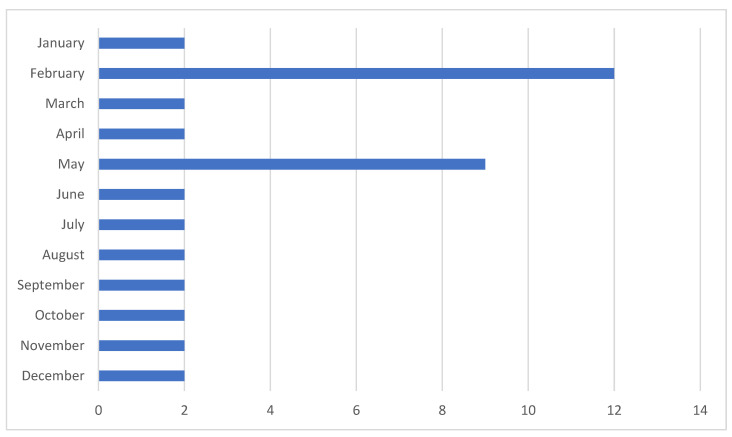
ADI frequency by month in 2023.

In 2023, the distribution of ADI cases by calendar month displayed two notable incidence peaks. The highest occurrence was recorded in February, with 12 out of 41 cases, followed by May, with 9 out of 41 cases. These peaks suggest potential temporal factors contributing to the increased rates of ADI during these months, such as school holidays or seasonal variations in supervision and exposure. Figure 2 illustrates these trends, highlighting the concentration of cases during these specific periods.

Table 4 provides a comprehensive summary of the medications most frequently implicated in ADI during the two years of the study, highlighting important trends in pharmaceutical exposure. Benzodiazepines, specifically diazepam, emerged as the most commonly involved drug, with a total of 25 cases (16 in 2022 and 9 in 2023), reflecting its widespread availability and potential misuse. Acetaminophen, known for its hepatotoxic potential, was the second most frequently reported agent, contributing to 19 cases (13 in 2022 and 6 in 2023), underscoring its risk when used inappropriately or unsupervised.

Antibiotics also featured prominently, collectively accounting for a substantial number of cases. Trimethoprim-sulfamethoxazole (Biseptol), amoxicillin, and cefuroxime were the leading antibiotics involved, with 14, 15, and 9 cases, respectively. These findings suggest that the accessibility and widespread use of antibiotics in pediatric settings may contribute to accidental or inappropriate exposure. Additionally, other medications, such as ibuprofen (11 cases) and cardiovascular agents such as furosemide and digoxin (4 cases each), highlight the diverse range of substances posing risks in ouseholds.

While less frequent, barbiturates such as phenobarbital (5 cases) and bronchodilators such as salbutamol (1 case in 2023) demonstrate the potential for intoxications across various therapeutic classes. These findings collectively emphasize the importance of safe medication storage, caregiver education, and targeted interventions to mitigate the risk of ADI in pediatric populations. No statistically significant changes in the distribution of specific medications between years were found (*p* > 0.05).

Structured patient interviews and exposure history assessments establish the timeline and identify the ingested substance, differentiating intentional from accidental cases. Physical examinations focus on neurological, cardiovascular, and respiratory assessments to detect toxicological effects.

Laboratory tests, including liver and renal panels, evaluate systemic organ damage, while imaging studies, such as abdominal X-rays and ultrasounds, help identify physical complications. Neurological monitoring, using EEG and reflex testing, assesses the severity of CNS toxicity, while cardiac monitoring (ECG and troponin levels) identifies arrhythmias and myocardial damage. Respiratory assessments evaluate oxygenation and acid–base imbalances caused by toxic substances.

Severity scoring systems, such as the PSS, classify cases into mild, moderate, and severe categories, with severe cases linked to higher doses of single toxic agents and delayed medical intervention. Delayed presentations were found to worsen outcomes, particularly in acetaminophen-induced hepatotoxicity. Table 5 emphasizes the importance of a multidisciplinary diagnostic framework for effective management.

The main clinical manifestations encountered in our study pertained to the nervous, digestive, and cardiac system, which were not characteristic of a specific type of drug. In diazepam intoxication, the following were recorded: somnolence: 11/25 patients; coma: 3/25 patients; balance disorders: 4/25 patients; headache: 4/25 patients; dizziness: 4/25 patients; psychomotor agitation: 1/25 patients; mydriasis: 4/25 patients; and bradycardia: 1/25 patients. The same clinical manifestations were recorded in other intoxications: abdominal pain and vomiting.

In relation to the severity of clinical forms, mild cases predominated in 2022 (53.2%), and in 2023, medium cases (42.9%); severe cases remained stable across both years (~29%) (Table 6). A statistically significant difference was found in the distribution of mild vs. moderate cases between years (*p* < 0.05).

The majority of cases involved drug ingestion, with a single exception in which a teenager inhaled a high dose of salbutamol. The mean time from drug ingestion to hospital admission was prolonged, primarily due to the transfer of some intoxicated children from remote areas, with an average duration of 4.8 h in 2022 and 5.5 h in 2023. However, the standard deviation substantially exceeded the mean, reaching 10.3 h in 2022 and 15.2 h in 2023, indicating significant variability in individual cases. The range of time intervals was notably wide, spanning from 0.3 to 72 h (Table 7).

Table 8 summarizes the types of medical interventions performed in cases of acute drug intoxication during 2022 and 2023, encompassing a total of 120 cases (79 in 2022 and 41 in 2023).

Supportive care alone was the most frequent intervention (44.2% of cases), followed closely by intravenous fluid administration, which was the most commonly implemented single intervention across both years, being used in 63.3% of cases in 2022 and 61.0% in 2023, with a total utilization rate of 62.5%. Antidote administration was employed in 34.2% of cases in 2022 and in 39.0% in 2023, accounting for 35.8% of the total cases.

Other interventions included activated charcoal (22.5% overall), gastric lavage (15.0% overall), and oxygen therapy (25.0% overall). Psychiatric support was provided in 26.7% of cases, reflecting the mental health challenges often associated with intoxication cases, while continuous monitoring in the intensive care unit (ICU) was required in 20.8% of the cases, indicating the severity of some incidents.

Interventions targeting specific complications, such as seizure management (10.8%) and respiratory support (29.2%), highlight the clinical need for rapid and specialized responses in certain cases. The similar proportions across the two years suggest a consistent approach to treatment strategies for acute drug intoxications. Intravenous fluids were the most common intervention (62.5%), followed by supportive care (44.2%), and antidote use increased slightly in 2023 (39%) compared to 2022 (34.2%), but this was not statistically significant (*p* > 0.05).

In 2022, the average hospitalization duration was 3.84 days, with a standard deviation of ±1.99 days, indicating moderate variability. The duration of hospitalization ranged from a minimum of 1 day to a maximum of 11 days.

In contrast, the average hospital stay in 2023 was significantly shorter at 2.32 days, with a lower standard deviation of ±0.93 days, reflecting less variation among cases. The range of hospitalization in 2023 was also narrower, spanning from 1 to 7 days. A significant reduction in mean hospital stay was observed from 2022 (3.84 ± 1.99 days) to 2023 (2.32 ± 0.93 days) (*p* < 0.05, odds ratio 0.29), suggesting improved management efficiency (Table 9).

In the cohort of 120 children with ADI, no fatalities were recorded. It is generally recognized that children respond more favorably to treatment compared to adults, largely due to the absence of comorbidities. This factor contributes to the lower mortality rates observed in pediatric poisoning cases. Upon discharge, families of children who had experienced unintentional poisoning were counseled on preventive measures, including the safe storage of medications out of the reach of children. For adolescents, parents were advised to maintain close communication and emotional support and to seek specialist consultations in pediatric psychology or psychiatry when necessary.

The prevention of unintentional ADI is achievable through appropriate education and awareness among family members and caregivers, emphasizing the importance of proactive measures to safeguard children.

### Practical Prevention Strategies

Based on the findings of our study, we propose a series of targeted recommendations aimed at mitigating the risk of pediatric acute drug intoxications. First, caregivers should be advised to store all medications in locked cabinets or containers positioned out of the reach and sight of children. The use of child-resistant packaging is imperative and should be emphasized as a routine safety measure, with instructions to securely close the packaging after each use.

Recognizing the early signs of drug intoxication is critical for timely intervention. Symptoms such as excessive drowsiness, unexplained vomiting, abdominal discomfort, and altered mental status should alert parents and caregivers to seek immediate medical assistance. Furthermore, efforts to educate caregivers about avoiding practices that could inadvertently encourage accidental ingestion, such as referring to medications as “candy”, are essential.

Families should also be encouraged to maintain readily available contact information for poison control centers and emergency services, ensuring rapid access in critical situations. Basic first-response education tailored to the common types of intoxication observed in pediatric populations should be disseminated during routine healthcare visits or through public health campaigns. This could include avoiding actions, such as inducing vomiting, unless explicitly directed by medical professionals.

Integrating these measures into community and healthcare settings could significantly reduce the incidence and severity of pediatric drug intoxications. Such preventive strategies align with our study’s findings and contribute to the development of a safer environment for children, addressing both accidental and intentional exposure.

## 4. Discussion

Our study identified a predominance of ADI among females (73.3%) and children from urban areas (77.5%), findings that echo other epidemiological studies. Gummin et al. similarly reported higher rates of poisoning in urban settings, which can be attributed to increased access to medications and household chemicals in densely populated areas. Female predominance in adolescents has been linked to higher rates of intentional ingestions, often associated with psychiatric comorbidities such as depression and anxiety [15]. In contrast, studies conducted in rural regions, such as Balme et al., noted an inverse trend, with higher rates of accidental poisoning due to agricultural chemicals, emphasizing the role of geographical context [16].

The age-related distribution in our study, with accidental ingestions predominant among children aged 1–5 years and voluntary ingestions among adolescents aged 10–17 years, reflects well-documented trends. Watson et al. highlighted that curiosity-driven exploratory behavior in younger children increases the risk of accidental ingestion, while adolescence introduces intentional poisoning as a significant concern, often linked to self-harm [17]. The voluntary ingestion rate (47.5% in adolescents) aligns with data from Zosel et al., who reported similar patterns globally [18].

Benzodiazepines (20.8%) were the leading cause of intoxication in our cohort, followed by acetaminophen and antibiotics. This finding corroborates the work of Lam, who identified similar trends in pediatric poisonings [19]. The predominance of neurological symptoms in benzodiazepine poisonings and the potential for severe outcomes, such as respiratory depression, underscores the critical need for timely diagnosis and administration of flumazenil. Benzodiazepine intoxications are a significant concern in pediatric toxicology due to its widespread availability and high potential for misuse. These substances, commonly prescribed for anxiety, insomnia, and seizures, have been consistently reported as a leading cause of acute intoxications in children and adolescents. Our study corroborates these findings, with benzodiazepines accounting for 20.8% of cases, making them the most frequently implicated drug category. Globally, studies have highlighted the dangers associated with benzodiazepine exposure in pediatric populations. Research from the United States, for instance, identified benzodiazepines as the second most common pharmaceutical agent involved in intentional poisonings among adolescents, frequently linked to self-harm or suicide attempts [1]. In Europe, a similar pattern has been observed, with benzodiazepines representing a substantial proportion of hospital admissions for acute drug toxicity in both accidental and intentional cases [2]. These trends are exacerbated by the ease of access to these medications, either through prescriptions or unmonitored household storage, particularly in urban settings, where availability is higher. The clinical manifestations of benzodiazepine intoxications are diverse, ranging from mild sedation to life-threatening respiratory depression and coma. Timely diagnosis and intervention are critical, with flumazenil serving as a specific antidote in severe cases. However, the use of flumazenil requires caution due to its potential to precipitate withdrawal seizures, particularly in chronic users or those with co-ingestants. This underscores the complexity of managing benzodiazepine poisonings and the necessity for healthcare professionals to remain vigilant.

The recurrent presence of benzodiazepines in intoxication cases also highlights broader issues of prescribing practices and medication safety. Educational initiatives aimed at healthcare providers and caregivers should emphasize the risks associated with benzodiazepine prescriptions, especially for families with adolescents. Additionally, public health strategies to improve safe storage and disposal practices are critical in mitigating accidental and intentional exposures.

Given the persistent burden of benzodiazepine intoxications in pediatric populations, further research is warranted to explore the socio-demographic factors influencing these patterns and to develop targeted prevention and intervention strategies. This issue must remain a priority for healthcare experts to ensure the safety and well-being of vulnerable groups.

Acetaminophen poisonings, implicated in 15% of cases in our study, continue to be a global challenge due to its potential for acute liver failure. Daly et al. emphasize the importance of early intervention with N-acetylcysteine to mitigate hepatotoxicity [20].

The notable presence of antibiotics in our study (46.7% of cases) differs from most literature, where non-pharmaceutical substances often dominate. This finding may reflect the prescribing practices and storage habits in our region. The role of antibiotics in pediatric ADI has been less studied, warranting further investigation into the clinical and toxicological implications. This prevalence likely reflects the high prescription rates and widespread availability of antibiotics in the region, coupled with inadequate storage practices in many households. Studies have shown that caregivers often store leftover antibiotics after treatment courses, believing that they might be useful for future illnesses, thereby increasing the risk of accidental ingestion by children [20]. Furthermore, the accessibility of over-the-counter antibiotics in some settings exacerbates the issue, as these drugs are often obtained without proper medical guidance.

The involvement of antibiotics in pediatric intoxications underscores the urgent need for public health interventions to address their misuse. These could include stricter regulations on the sale and distribution of antibiotics, ensuring that they are dispensed only with valid prescriptions. Healthcare providers play a critical role in educating families about the importance of completing prescribed courses and safely disposing of unused medications. Awareness campaigns emphasizing the risks associated with antibiotic misuse, both in terms of toxicity and the broader implications of antimicrobial resistance, should also be prioritized. Additionally, implementing medication storage education as part of routine pediatric consultations can significantly reduce accidental exposure. Caregivers should be encouraged to store antibiotics in locked or child-resistant containers, placed out of the reach and sight of children. These strategies, combined with continued efforts to optimize prescribing practices and limit unnecessary antibiotic use, can mitigate the risks associated with these medications in pediatric populations [21].

Seasonal trends in our data revealed peaks in March and August 2022 and in February and May 2023, which could be linked to school holidays and increased unsupervised time for children. Jolliff et al. similarly reported seasonal spikes in poisoning cases during school vacations, highlighting the need for heightened preventive measures during these periods [22]. These trends have been similarly reported in studies from South Africa and Southeast Asia, where the absence of structured activities during school breaks often leads to increased unsupervised time, thereby elevating the risk of accidental exposure to medications and other toxic substances. Additionally, changes in weather and the increased prevalence of illnesses during certain months may contribute to higher household medication usage, inadvertently increasing accessibility for children. For example, antibiotics and antipyretics, which were frequently implicated in our study, are often prescribed more frequently during flu seasons, potentially explaining their prominence in intoxication cases during these months. To address these seasonal risks, specific preventive strategies should be implemented during high-risk periods. Public health campaigns targeting caregivers could emphasize the importance of secure medication storage and increased supervision during school holidays. Educational initiatives within schools could also raise awareness among children and adolescents about the dangers of unsupervised medication use. Moreover, healthcare providers should proactively counsel families during peak prescription seasons on safe medication practices, including the proper disposal of expired or unused drugs. Establishing community-based poison prevention programs that provide resources and education tailored to these seasonal patterns could further mitigate risks [23].

In our cohort, mild cases predominated (44.2%), while severe cases accounted for 29.2%. The severity distribution aligns with the Poisoning Severity Score framework proposed by Persson et al., which categorizes cases based on clinical outcomes [24]. Notably, moderate cases increased in 2023 (42.9%), suggesting potential changes in exposure patterns or healthcare-seeking behavior.

Respiratory support was required in 29.2% of cases, with 15% necessitating mechanical ventilation. This aligns with data from Lee et al., who emphasize the importance of early respiratory assessment in severe poisonings. The overall use of antidotes (35.8%) and activated charcoal (22.5%) in our study is consistent with international guidelines, which advocate targeted interventions for specific toxins [8]. The mean hospitalization duration in our study was relatively short (3.8 ± 1.9 days in 2022 and 2.3 ± 0.9 days in 2023), reflecting efficient management practices and the predominance of mild cases. The absence of fatalities aligns with the general trend reported in pediatric ADI, where mortality rates are low, provided timely medical care is accessible. However, disparities in healthcare infrastructure can significantly impact these outcomes in resource-limited settings [1].

Our findings underscore the need for multifaceted preventive strategies. Enhanced parental education on medication storage, particularly in urban settings, remains paramount. Additionally, school-based awareness programs targeting adolescents may help address the psychosocial factors contributing to intentional poisonings. Community-level poison control initiatives and telemedicine platforms could further support early intervention and reduce the burden on healthcare facilities.

Further research is warranted to explore the role of socioeconomic factors, cultural practices, and healthcare accessibility in shaping ADI trends. Comparative studies across regions with varying healthcare resources could provide valuable insights into optimizing prevention and treatment strategies. The findings of our study align with global trends in pediatric acute drug intoxications (ADIs), while also highlighting regional specifics. For instance, our observation that accidental intoxications are more prevalent in children aged 1–5 years mirrors reports from studies conducted in Bangladesh and Romania, where this age group accounted for 60–80% of ADI cases due to unsupervised access to medications in households [5,20]. Similarly, the high rate of intentional ingestions among adolescents in our cohort (47.5%) is consistent with data from international studies, such as those conducted in the United States and Australia, where self-harm behaviors and mental health challenges were predominant drivers of adolescent poisonings [25].

In terms of substance involvement, benzodiazepines and acetaminophen were among the leading agents in our study, a finding echoed by research from Europe and North America, where these drugs are commonly implicated in pediatric poisonings due to their widespread availability [8,15]. However, the high prevalence of antibiotic-related intoxications in our cohort (46.7%) diverges from trends observed in studies from high-income countries, where non-pharmaceutical substances, such as household chemicals and plants, often dominate. This discrepancy may reflect differences in prescribing practices, medication storage habits, and public health education between regions.

The seasonal peaks observed in our study, particularly during school holidays, are consistent with findings from South Africa and Southeast Asia, where similar temporal patterns were attributed to reduced supervision and changes in daily routines during these periods [26,27]. These cross-regional similarities and differences underscore the multifaceted nature of pediatric ADIs and highlight the need for tailored prevention strategies that address local risk factors while drawing on international best practices.

This study highlights the significant burden of ADIs in pediatric populations, with findings that are consistent with global trends while offering specific regional insights. The predominance of cases among females, children from urban areas, and those in the 1–5 year and 10–17 year age groups underscores the need for targeted prevention efforts. The high prevalence of benzodiazepine-related intoxications, followed by acetaminophen and antibiotics, reflects the regional availability and usage patterns of these substances. These findings point to the necessity of stricter medication storage practices and improved public awareness to mitigate risks, particularly for easily accessible drugs. Clinically, the predominance of neurological symptoms, especially in benzodiazepine poisonings, and the substantial proportion of severe cases requiring intensive interventions, such as respiratory support and ICU monitoring, emphasize the importance of timely diagnosis and effective management. While the absence of fatalities underscores the efficacy of current treatment protocols, the frequent need for critical care highlights the potential severity of these cases and the strain they can place on healthcare resources. In addition, the role of antidote administration and activated charcoal in reducing morbidity highlights the value of adhering to evidence-based treatment protocols. This study has several limitations that impact the interpretation of its findings. The retrospective design relies on medical records, which may be incomplete, thereby introducing potential inaccuracies. Biases, such as underreporting in rural areas and selection bias from focusing on hospitalized cases, may skew the results toward more severe intoxications. The exclusion of non-pharmaceutical substances and mixed poisonings limits generalizability, while disparities in urban and rural case distributions may reflect differences in healthcare access rather than true incidence. Additionally, the two-year study period does not capture long-term trends, and variability in diagnostic methods may have influenced case confirmation. Future prospective studies with larger, more diverse populations and longer observation periods are needed to address these gaps.

## 5. Conclusions

In conclusion, this study highlights the significant burden of pediatric acute drug intoxications, with distinct patterns of accidental ingestions in younger children and intentional overdoses in adolescents. Benzodiazepines, acetaminophen, and antibiotics were the most frequently implicated substances. The findings emphasize the importance of targeted preventive strategies, including parental education, adolescent awareness programs, and improved rural healthcare access. While the reduction in hospitalization duration reflects advances in clinical management, the substantial proportion of moderate to severe cases underscores the need for ongoing improvements in prevention and treatment.

## Figures and Tables

**Table 1:** Frequency of AMI by gender, environment of origin, and age groups.

		Total (Number, Percentage)		2022 (Number, Percentage)		2023 (Number, Percentage)		*p*-Value
Gender	M	32	26.7%	23	71.9%	9	28.1	0.532
	F	88	73.3%	56	63.6%	32	36.4%	
Living environment	U	93	77.5%	61	65.6%	32	34.3%	1
	R	27	22.5%	18	66.7%	9	33.3%	
Age group (years)	0–1	3	2.5%	1	33.3%	2	66.7%	0.302
	1–5	54	45%	42	77.8%	12	22.2%	0.014
	6–9	6	5%	4	66.7%	2	33.3%	0.972
	10–17	57	47.5%	32	56.1%	25	43.9%	0.038

**Table 2:** Distribution of ADI by sex and environment of origin.

Study Year	Total	Urban		Rural	
		Masculine	Feminine	Masculine	Feminine
2022	79	17 (21.52%)	44 (54.69%)	6 (7.6%)	13 (16.45%)
2023	41	8 (19.51%)	24 (58.54%)	1 (2.44%)	7 (17.07%)
Total	120	25 (20.83%)	68 (56.66%)	7 (5.83%)	20 (16.6%)

**Table 3:** Comprehensive family and socioeconomic data of pediatric patients.

	Variable	2022	2023	Total
Socioeconomic Status	Low income	36	19	55
	Middle income	33	18	51
	High income	10	4	14
Parental Employment Status	Both parents employed	47	24	71
	Only one parent employed	21	12	33
	Both parents unemployed	11	5	16
Parental Education Level	Primary education	32	17	49
	Secondary education	31	16	47
	Tertiary education	16	8	24
Number of Siblings in Household	No siblings	8	3	11
	1–2 siblings	41	20	61
	3 or more siblings	30	18	48
Medication Accessibility in Household	Medications stored in secured locations	45	22	67
	Medications stored in unsecured locations	34	19	53
History of Chronic Illness in the Family	Positive history	12	8	20
	No chronic illness history	67	33	100

**Table 4:** Distribution of drug types involved in acute intoxications across 2022 and 2023.

No.	Medicine Type	2022	2023	Total
1	Benzodiazepines: Diazepam	16	9	25
2	Barbiturates: Phenobarbital	3	2	5
3	Antipyretics: Acetaminophen	13	6	19
4	Analgesics/Antipyretics: Ibuprofen	6	5	11
5	Diuretics: Furosemide	3	1	4
6	Cardiotonic agents: Digoxin	3	1	4
7	Antibiotics: Trimetoprim-Sulphamethoxazole	8	5	14
8	Antibiotics: Amoxicillin	11	4	15
9	Antibiotics: Cefuroxime	6	3	9
10	Antibiotics: Rifampicin	3	1	4
11	Antibiotics: Levofloxacin	7	3	10
12	Bronchodilators: Salbutamol inhaler	0	1	1

**Table 5:** Comprehensive diagnostic and monitoring approaches for acute drug intoxications.

Category	Test/Method	Purpose	Observation
Clinical History and Anamnesis	Structured Patient and Family Interview Exposure History Analysis: Timing, dose, and type of substance ingested.	Establish exposure timeline, identify likely agents, and differentiate intentional vs. accidental ingestion.	Parents frequently reported accidental ingestion of benzodiazepines or antibiotics; adolescents indicated intentional ingestion of acetaminophen or ibuprofen.
Physical Examination	**Neurological Assessment**: Glasgow Coma Scale (GCS), seizure activity. **Cardiovascular Evaluation**: Heart rate, blood pressure. **Respiratory Function**: Oxygen saturation, respiratory rate.	Detect signs indicating specific toxicants and assess severity.	Reduced GCS in diazepam cases; tachycardia and respiratory alkalosis noted in salbutamol inhaler intoxications.
Metabolic Testing	**Liver Function Tests (ALT, AST, INR)**: Assessed hepatotoxic effects of acetaminophen, rifampicin. **Renal Function Panel (Creatinine, BUN)**: Evaluated nephrotoxicity from NSAIDs, antibiotics.	Monitor systemic organ damage caused by ingested substances.	ALT levels > 2× normal confirmed acetaminophen hepatotoxicity; creatinine elevation (>1.5 mg/dL) indicated renal impairment in antibiotic-related intoxications.
Imaging Studies	**Abdominal X-Ray**: Identified radiopaque substances or foreign bodies. **Ultrasound**: Evaluated liver and kidney for structural damage.	Detect physical evidence of ingestion or complications.	No radiopaque substances identified; ultrasounds ruled out mechanical obstructions and organ enlargement in all cases.
Neurological Monitoring	**Electroencephalogram (EEG)**: Monitored seizures or CNS depression. **Pupil Reflex and Motor Assessment**: Checked for CNS involvement.	Evaluate the severity of CNS toxicity, such as seizures or coma.	Seizure activity recorded in barbiturate overdoses; reduced reflexes observed in diazepam-related toxicity.
Cardiac Monitoring	**Electrocardiogram (ECG)**: Identified arrhythmias or conduction abnormalities. **Troponin Levels**: Detected myocardial damage in severe cases.	Monitor cardiotoxic effects of drugs like digoxin, salbutamol, or tricyclic antidepressants.	QT prolongation and bradycardia noted in digoxin cases; tachycardia linked to salbutamol inhaler toxicity.
Respiratory Assessment	**Pulse Oximetry**: Measured oxygen saturation. **Capnography and Arterial Blood Gas (ABG)**: Assessed respiratory function and acid-base balance.	Detect respiratory depression or hyperventilation caused by toxic substances.	Oxygen saturation <90% indicated respiratory depression in CNS depressant cases; ABG revealed respiratory alkalosis in salbutamol toxicity.
Severity Scoring	**PSS**: Mild, moderate, severe classification.	Assess overall severity and predict outcomes.	Severe cases accounted for 29.2% of the cohort and were associated with delayed presentation or higher doses of a single toxic agent. Mild and moderate cases were effectively managed with supportive care.
Time from Ingestion	Time Analysis: Documented delay between ingestion and medical intervention.	Established prognosis and timing of antidote therapy.	Longer delays (>6 h) correlated with increased severity in acetaminophen-related hepatotoxicity.

**Table 6:** Clinical forms of ADI by years of study.

		Clinical Form		
Year of Study	Total	Mild	Medium	Severe
2022	79	42 (53.16%)	14 (17.72%)	23 (29.1%)
2023	41	11 (26.83%)	18 (43.9%)	12 (29.27%)
Total	120	53 (44.17%)	32 (26.62%)	35 (29.16%)

**Table 7:** Mean duration +/− standard deviation from ingestion to admission.

Year	Number of Patients	Average Duration (Hours)	Standard Deviation	Extremes (Hours)
2022	79	4.87	±10.336	0.5–72
2023	41	6.86	±17.206	0.3–48

**Table 8:** Types of interventions in acute drug intoxication (2022–2023).

Type of Intervention	Number of Patients in 2022	Number of Patients in 2023	Total
Supportive Care Only	35 (44.3%)	18 (43.9%)	53 (44.2%)
Antidote Administration	27 (34.2%)	16 (39.0%)	43 (35.8%)
Gastric Lavage	12 (15.2%)	6 (14.6%)	18 (15.0%)
Activated Charcoal	18 (22.8%)	9 (22.0%)	27 (22.5%)
Intravenous Fluids	50 (63.3%)	25 (61.0%)	75 (62.5%)
Oxygen Therapy	20 (25.3%)	10 (24.4%)	30 (25.0%)
Seizure Management	8 (10.1%)	5 (12.2%)	13 (10.8%)
Respiratory Support	22 (27.8%)	13 (31.7%)	35 (29.2%)
Psychiatric Support	20 (25.3%)	12 (29.3%)	32 (26.7%)
Continuous Monitoring in ICU	15 (19.0%)	10 (24.4%)	25 (20.8%)

**Table 9:** Average length of hospital stay +/− standard deviation per year of study.

Year	Number of Patients	Average Duration (Days)	Standard Deviation	Extremes (Days)
2022	79	3.84	±1.99	1–11
2023	41	2.32	±0.93	1–7

## Data Availability

Data are contained within the article.
